# Antibody-dependent cellular cytotoxicity against SARS-CoV-2 Omicron sub-lineages is reduced in convalescent sera regardless of infecting variant

**DOI:** 10.1016/j.xcrm.2022.100910

**Published:** 2022-12-22

**Authors:** Simone I. Richardson, Prudence Kgagudi, Nelia P. Manamela, Haajira Kaldine, Elizabeth M. Venter, Thanusha Pillay, Bronwen E. Lambson, Mieke A. van der Mescht, Tandile Hermanus, Sashkia R. Balla, Zelda de Beer, Talita R. de Villiers, Annie Bodenstein, Gretha van den Berg, Marizane du Pisanie, Wendy A. Burgers, Ntobeko A.B. Ntusi, Fareed Abdullah, Veronica Ueckermann, Theresa M. Rossouw, Michael T. Boswell, Penny L. Moore

**Affiliations:** 1 National Institute for Communicable Diseases of the National Health Laboratory Services, Johannesburg, South Africa; 2 South African Medical Research Council Antibody Immunity Research Unit, School of Pathology, University of the Witwatersrand, Johannesburg, South Africa; 3 Department of Immunology, Faculty of Health Sciences, University of Pretoria, Pretoria, South Africa; 4 Tshwane District Hospital, Pretoria, South Africa; 5 Division for Infectious Diseases, Department of Internal Medicine, Steve Biko Academic Hospital and University of Pretoria, Pretoria, South Africa; 6 Institute of Infectious Disease and Molecular Medicine, University of Cape Town, Cape Town, South Africa; 7 Division of Medical Virology, Department of Pathology; University of Cape Town, Cape Town, South Africa; 8 Wellcome Centre for Infectious Disease Research in Africa, University of Cape Town, Cape Town, South Africa; 9 Department of Medicine, University of Cape Town and Groote Schuur Hospital, Cape Town, South Africa; 10 Hatter Institute for Cardiovascular Research in Africa, Faculty of Health Sciences, University of Cape Town, Cape Town, South Africa; 11 Centre for the AIDS Programme of Research in South Africa, Durban, South Africa

**Keywords:** Omicron BA.4, antibody-dependent cellular cytotoxicity, neutralization, SARS-CoV-2, breakthrough infection, COVID-19, VOC, Fc effector function

## Abstract

The severe acute respiratory syndrome coronavirus 2 (SARS-CoV-2) Omicron BA.4 and BA.5 variants caused major waves of infections. Here, we assess the sensitivity of BA.4 to binding, neutralization, and antibody-dependent cellular cytotoxicity (ADCC) potential, measured by FcγRIIIa signaling, in convalescent donors infected with four previous variants of SARS-CoV-2, as well as in post-vaccination breakthrough infections (BTIs) caused by Delta or BA.1. We confirm that BA.4 shows high-level neutralization resistance regardless of the infecting variant. However, BTIs retain activity against BA.4, albeit at reduced titers. BA.4 sensitivity to ADCC is reduced compared with other variants but with smaller fold losses compared with neutralization and similar patterns of cross-reactivity. Overall, the high neutralization resistance of BA.4, even to antibodies from BA.1 infection, provides an immunological mechanism for the rapid spread of BA.4 immediately after a BA.1-dominated wave. Furthermore, although ADCC potential against BA.4 is reduced, residual activity may contribute to observed protection from severe disease.

## Highlights

• Omicron BA.4 escapes neutralization and ADCC elicited by infection with VOCs
• BA.4 sensitivity to ADCC is reduced compared with other VOCs
• ADCC shows smaller fold losses compared with neutralizing antibodies
• Residual ADCC activity may contribute to protection from severe disease

## Graphical abstract

**Figure:**
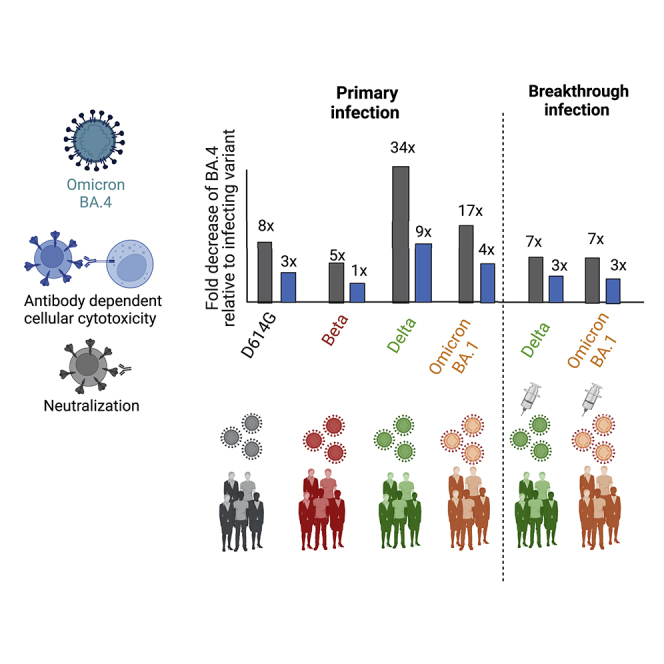

## Teaser

Richardson et al. show that SARS-CoV-2 Omicron BA.4 not only evades the ability of antibodies to neutralize but also reduces their potential to lyse infected cells, albeit to a lesser extent. Despite this reduction, residual ADCC activity may nonetheless contribute to the protection from severe disease.

## Introduction

The emergence of severe acute respiratory syndrome coronavirus 2 (SARS-CoV-2) variants of concern (VOCs) bearing mutations in the spike protein has resulted in escape from neutralizing antibodies (nAbs) triggered by vaccination and infection^1^^,^^2^^,^^3^^,^^4^^,^^5^^,^^6^^,^^7^ and subsequently reduced protection from infection.^8^^,^^9^ These VOCs include Omicron BA.1, containing over 30 mutations in the spike region, against which neutralization titers are further reduced.^10^^,^^11^ In contrast, the ability of vaccines to prevent severe disease has been maintained.^9^^,^^12^^,^^13^ This is likely due to the preserved activity of T cells and Fc effector function, including antibody-dependent cellular cytotoxicity (ADCC), against VOCs.^14^^,^^15^^,^^16^^,^^17^^,^^18^^,^^19^ While the correlates of protection after vaccination are incompletely understood, neutralization, T cells, and Fc effector function have all been suggested to play an important role.^20^^,^^21^^,^^22^ Specifically, Fc effector function has been associated with reduced COVID-19 mortality and severity,^23^ is required for monoclonal antibodies to optimally protect from infection,^24^^,^^25^ and correlates with vaccine protection in animal models.^20^^,^^26^^,^^27^^,^^28^ As a result, understanding the impact of VOCs on Fc effector function is likely key for vaccine design.

Omicron has since evolved into several sub-lineages.^29^^,^^30^ The BA.4 and BA.5 sub-lineages, which share the same spike sequence but differ from one another in non-structural protein and membrane (M) genes, drove the fifth wave of infection in South Africa and were responsible for significant numbers of infections in several other countries.^30^ BA.4 and BA.5 are genetically similar to BA.2 but contain two additional mutations in the receptor-binding domain (RBD), L452R and F486V. As a consequence, compared with BA.1 and BA.2, BA.4 has shown increased neutralization resistance to convalescent sera, vaccinee sera, and monoclonal antibodies.^6^^,^^31^^,^^32^^,^^33^ However, the effect of sub-lineages beyond BA.1 on Fc effector function is unknown.^17^^,^^34^

We and others have shown that each SARS-CoV-2 variant triggers different profiles of nAbs and Fc effector function.^16^^,^^18^^,^^35^^,^^36^^,^^37^ For example, the Beta variant triggered humoral responses with increased cross-reactivity, whereas Omicron triggered more strain-specific nAbs.^16^^,^^18^^,^^35^^,^^36^ Here, we assessed the sensitivity of BA.1 and BA.4 to nAbs and ADCC potential as measured by FcγRIIIa signaling (but hereafter referred to as ADCC) elicited by infections caused by D614G, Beta, Delta, or BA.1 (responsible for the first four waves in South Africa) in vaccinated and unvaccinated individuals.

We confirm that BA.4 shows high-level resistance to neutralization regardless of the infecting variant. However, high neutralizing titers associated with breakthrough infection with either Delta or BA.1 after vaccination result in preserved neutralization against BA.4. Further, we show that while ADCC activity against BA.4 was reduced further than previously reported for other VOCs, it remained detectable in both convalescent plasma and in vaccine breakthrough infections. Overall, this study confirms the increased neutralization resistance of BA.4 and provides an immunological mechanism for the rapid spread of BA.4 in South Africa despite high levels of infections by previous VOCs.^38^ Furthermore, despite the reduced ADCC against BA.4, the residual activity we detect in convalescent plasma and vaccinees may nonetheless have contributed to the protection from severe disease.

## Results

### BA.4 escapes convalescent plasma neutralization regardless of the infecting strain

We assayed plasma from individuals infected in the first four waves of infection in South Africa, with D614G (wave 1, n = 16), Beta (wave 2, n = 10), Delta (wave 3, n = 7), or Omicron BA.1 (wave 4, n = 20), with clinical and demographic details presented in Table S1. Gender was not controlled for across the waves. All samples were obtained from individuals who reported no prior infection or vaccination, which was confirmed by national databases that are linked to individual national identification numbers.^18^^,^^35^ These samples were collected at a median of 3 days (range 1–11 days) post-infection with D614G, Delta, or BA.1 and a median of 14 days (range 8–29 days) post-infection with Beta.

Given the significant variation in the timing of sampling across the cohorts, we first measured antibody binding to the matched infecting variant spike by ELISA to assess whether there were significant differences in antibody levels that may be attributed to sampling bias. We found that there were no differences in autologous antibody levels across the cohorts (Figure S1).

**Figure 1:**
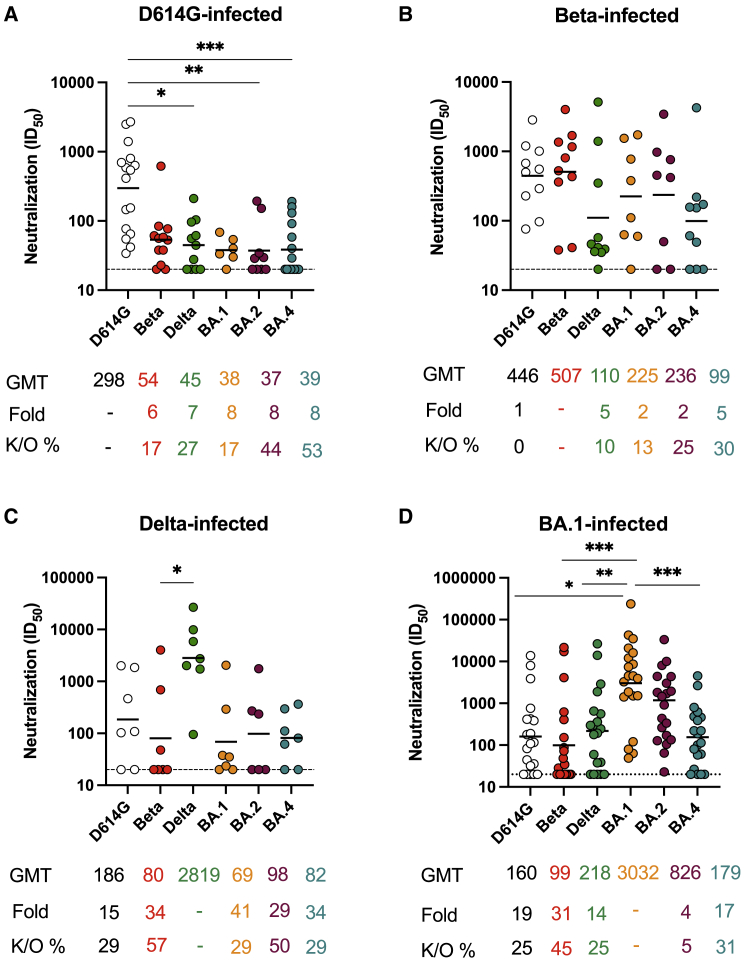
BA.4 neutralization escape varies by the infecting variant in unvaccinated convalescent individuals Pseudovirus neutralization titer (ID_50_) in convalescent plasma from unvaccinated donors infected with (A) D614G, (B) Beta, (C) Delta, and (D) Omicron BA.1. Plasma was tested against D614G, Beta, Delta, and Omicron BA.1, BA.2, and BA.4. Lines indicate geometric mean titer (GMT) also represented below the plot with fold decrease and knockout (KO) of detectable activity for other variants as a percentage relative to the infecting strain. Dotted lines indicate the limit of detection of the assay. Statistical significance across variants is shown by Friedman test with Dunn’s correction. ∗p < 0.05, ∗∗p < 0.01, and ∗∗∗p < 0.001. All data are representative of two independent experiments containing a minimum of two biological replicates.

Next, we measured pseudovirus neutralization activity across the four waves. Historical data were used for BA.1- and Delta-infected samples as previously described.^18^^,^^35^ Overall, we show that plasma from all infection waves exhibited decreased neutralization against BA.4, similar to BA.1 and BA.2 fold losses regardless of the infecting strain, with titers ranging from a geometric mean titer (GMT) of 39 in D614G infections to 179 in BA.1 infection (Figure 1). However, the fold loss of neutralization activity varied by wave. In D614G and Beta infections, where autologous titers against the infecting strains were lower, around <1:600, the loss in neutralization against BA.4 was 5- to 8-fold (Figures 1A and 1B), while Delta and BA.1 infections (both of which triggered high titers of >1:2,500 against their matched spikes, perhaps a consequence of higher viral loads) showed 34- and 17-fold losses against BA.4, respectively (Figures 1C and 1D). We also observed variant-specific differences in neutralization of Omicron BA.2, which showed similar titers to BA.4 in D614G and Delta infection but different titers in Beta and BA.1 infections (with BA.2 significantly more sensitive than BA.4, with titers of 826 and 179, respectively). In general, when considering the degree of cross-reactivity of antibodies triggered by each variant against multiple VOCs, we observed a greater number of significant fold losses for antibodies triggered by D614G (significant losses against Delta, BA.2, and BA.4) and by BA.1 (with significant fold losses against all variants except BA.2), as previously reported (Figures 1A and 1D).^18^ In contrast, Beta-elicited nAbs showed greater levels of cross-reactivity than those triggered by other variants, with no significant fold differences, as we have described elsewhere (Figure 1B).^36^ While Delta-infected plasma showed large fold differences between autologous responses and all other variants, only neutralization against Beta was significantly poorer as also previously described.^35^

### Breakthrough infection following vaccination shows increased neutralization cross-reactivity against BA.4

We next tested the capacity of plasma from breakthrough infections (BTIs) caused by Delta (n = 17) or Omicron BA.1 (n = 6, as also described elsewhere^18^) following vaccination^18^^,^^39^ to neutralize BA.4. Plasma samples obtained were collected at a median of 30 and 5 days for Delta and BA.1 BTIs, respectively, and were examined following either one dose of Ad26.CoV.2 or two doses of BNT162b2. The interval between vaccination and BTI was a median of 152 and 102 days for Delta and BA.1 infections, respectively. Similar to the infection waves, Delta and BA.1 BTI binding responses to the autologous infecting spike were not significantly different (Figure S1).

**Figure 2:**
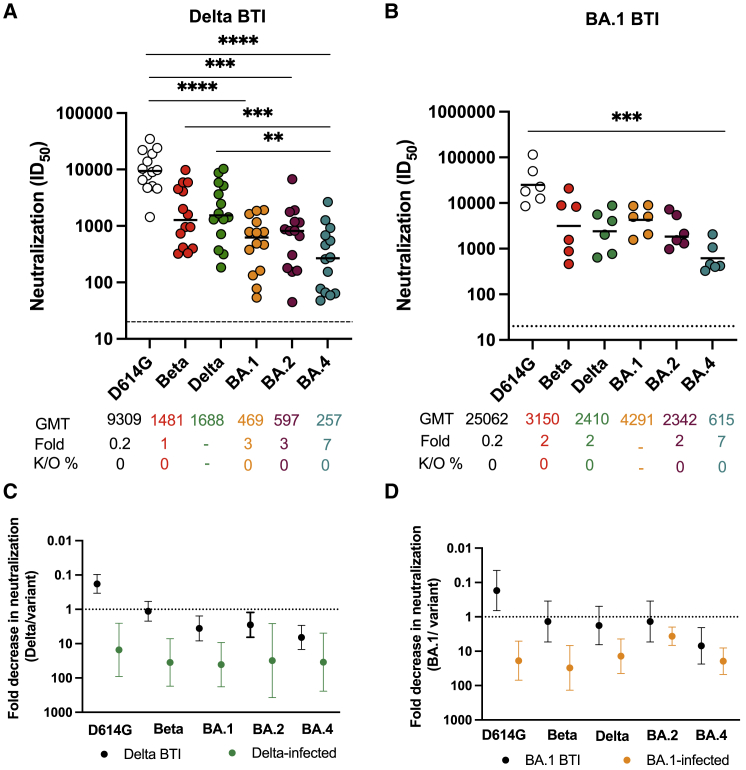
Breakthrough infections show reduced neutralization activity against BA.4 despite high titers against other VOCs (A and B) Pseudovirus neutralization titer (ID_50_) in convalescent plasma from vaccinated donors subsequently infected with (A) Delta and (B) Omicron BA.1. Plasma were tested against D614G, Beta, Delta, and Omicron BA.1, BA.2, and BA.4. Lines indicate GMT also represented below the plot with fold decrease and KO of activity for other variants as a percentage relative to the infecting variant. Dotted lines indicate the limit of detection of the assay. (C and D) Fold decrease in neutralization for each VOC represented as a ratio of the titer to the infecting variant Delta (C) or BA.1 (D) for infections in unvaccinated individuals (green for Delta and orange for BA.1) and BTIs (black). Dots represent mean fold decrease while error bars represent standard deviation of the mean. Statistical significance across variants is shown by Friedman test with Dunn’s correction. ∗∗p < 0.01, ∗∗∗p < 0.001, and ∗∗∗∗p < 0.0001. All data are representative of two independent experiments containing a minimum of two biological replicates.

We and others have previously shown that BTIs trigger high levels of nAbs that are cross-reactive for VOCs.^18^^,^^39^^,^^40^ To reflect this, BA.1 BTIs showed significantly higher binding levels compared with BA.1-unvaccinated plasma (Figure S1); however, Delta did not show the same trend. Historical neutralization data was used for BA.1 BTI samples as previously described.^18^ In both Delta and BA.1 BTIs, neutralization titers were highest against D614G (which matches the vaccine strain) rather than the infecting variant (Figures 2A and 2B). In Delta BTIs, titers against D614G, Beta, and Delta were higher than all Omicron sub-lineages (Figure 2A). For BA.1 BTIs, significant fold losses compared with D614G were only observed in BA.4, perhaps as a result of the small sample size (Figure 2B). Unlike previous VOCs, BA.4 shows substantially increased resistance to neutralization in BTIs caused by either Delta or Omicron BA.1. In Delta and BA.1 BTIs, we saw a 7-fold reduction in titers compared with titers against the infecting variant (Figures 2A and 2B), and in contrast to unvaccinated individuals, all samples retained neutralization activity against BA.4 (Figure 2B).

We next compared convalescent plasma from unvaccinated individuals with BTIs by the same variant to assess whether similar fold losses were observed in both cases (Figures 2C and 2D). In both Delta and BA.1, enhanced titers were observed against D614G, consistent with prior exposure to the vaccine sequence (ancestral strain), whereas all other ratios were >1, indicating decreased neutralization relative to the infecting variant. However, for Delta and BA.1, the fold decrease in neutralization against each variant was higher in unvaccinated individuals (Figures 2C and 2D, green and orange) compared with BTIs (Figures 2C and 2D, black). This suggests that hybrid immunity results in more resilient neutralizing responses to VOCs.

### BA.4 shows increased escape from ADCC potential compared with other VOCs

We next assessed the ability of plasma antibodies from convalescent donors from each of the four waves to cross-link D614G, Beta, Delta, BA.1, BA.2, or BA.4 cell-surface-expressed spike and activate FcγRIIIa (CD16) as a proxy for ADCC activity. This assay has been found to be a robust comparator to natural killer (NK) activation assays by others and has been used to define convalescent samples with broad activity profiles for potential infusion.^41^^,^^42^ Transiently transfected cell-surface spike levels were determined across variants by the binding of an antibody that targets soluble spike protein equivalently across VOCs (Figure S2). Additionally, using pre-pandemic controls, we determined a threshold for this assay as shown in Figure S3.

**Figure 3:**
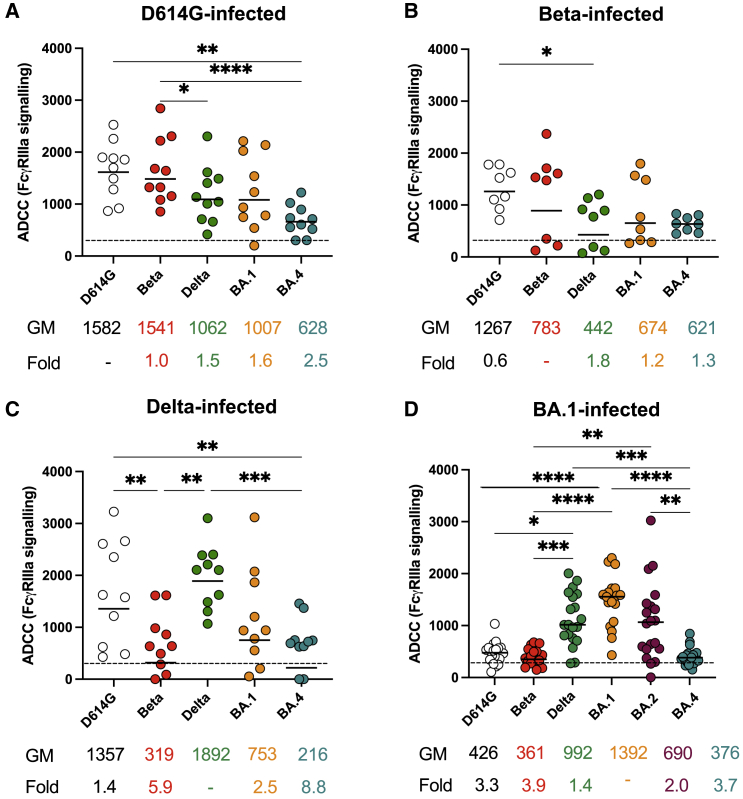
FcγRIIIa signaling (ADCC) against BA.4 is reduced but preserved in convalescent plasma from previously unvaccinated individuals regardless of the infecting variant Antibody-dependent cellular cytotoxicity (ADCC) in unvaccinated individuals infected with (A) D614G, (B) Beta, (C) Delta, and (D) Omicron BA.1. The ability of plasma to cross-link spike expressed on the surface of HEK293T cells and activate FcγRIIIa represented as normalized relative light units (RLU) with background as determined in the absence of antibody are shown. All data are representative of two independent experiments containing a minimum of two biological replicates. Lines indicate geometric mean (GM) RLUs, also represented below the plot with fold decrease of activity for other variants relative to the infecting variant. Statistical significance across variants is shown by Friedman test with Dunn’s correction. ∗p < 0.05, ∗∗p < 0.01, ∗∗∗p < 0.001, and ∗∗∗∗p < 0.0001.

As we have previously reported,^16^ fold loss in activity for ADCC was generally in the order of 2- to 3-fold, much less than for neutralization, likely due to the higher number of epitopes recognized on the spike. However, compared with ADCC against the matched spike in each wave, we observed 2- to 8.8-fold reduced activity against BA.4 (Figures 3A–3D). These losses were statistically significant, with the exception of Beta-triggered ADCC (Figure 3B), consistent with our previous studies suggesting that Beta triggers more cross-reactive ADCC.^16^ Compared with BA.1, BA.4 trended toward lower ADCC potential in plasma from all four waves but only significantly so in the BA.1-infected plasma. Other wave-specific patterns were observed such as significant decreases in ADCC of D614G-infected plasma against Delta compared with Beta (Figure 3A), of Beta-infected plasma against D614G compared with Delta (Figure 3B), and of Delta-infected plasma against Delta compared with Beta and D614G compared with both BA.4 and Beta (Figure 3C). Finally, for BA.1-infected plasma, in addition to BA.4, D614G and Beta were significantly lower than BA.1, BA.2, and Delta (Figure 3D). These observations mirrored neutralization but were significant for ADCC, likely as a result of the inclusion of more samples (in the case of Delta-infected plasma). Thus, ADCC activity against BA.4 in convalescent plasma from unvaccinated individuals was reduced compared with the infecting variant.

### ADCC potential elicited by Delta and Omicron BA.1 BTIs is compromised by BA.4

**Figure 4:**
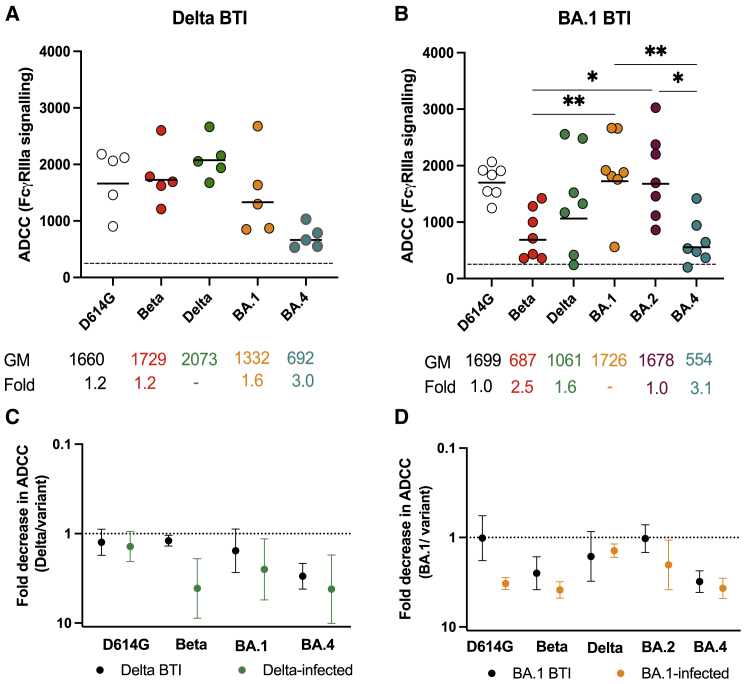
FcγRIIIa signaling (ADCC) against BA.4 is reduced in BTIs caused by Delta or BA.1 to a similar extent as in convalescent plasma (A and B) ADCC of vaccinated donors subsequently infected with (A) Delta and (B) Omicron BA.1. The ability of plasma to cross-link spike expressed on the surface of HEK293T cells and activate FcγRIIIa represented as normalized RLUs without background as determined in the absence of antibody are shown. Lines indicate GM RLUs, also represented below the plot with fold decrease of activity for other variants relative to the infecting variant. Positivity threshold is indicated with a dotted line as determined by pre-pandemic SARS-CoV-2-negative controls. (C and D) Fold decrease in ADCC for each VOC represented as a ratio of ADCC activity to the infecting variant Delta (C) or BA.1 (D) for infections in unvaccinated individuals (green for Delta and orange for BA.1) and BTIs (black). Dots represent mean fold decrease while error bars represent standard deviation of the mean. Statistical significance across variants is shown by Friedman test with Dunn’s correction. ∗p < 0.05 and ∗∗p < 0.01. All data are representative of two independent experiments containing a minimum of two biological replicates.

Using a sub-set of samples tested against neutralization, we measured FcγRIIIa activation for BTIs caused by Delta (n = 5) and BA.1 (n = 7) (Figures 4A and 4B). In line with what we have previously reported,^18^^,^^39^ ADCC activity was higher in individuals who were previously vaccinated and then infected compared with those who were not, regardless of the infecting variant. This included higher activity against BA.4 where normalized relative light units (RLUs) were 3.2-fold higher in Delta BTIs compared with Delta-infected unvaccinated individuals (p < 0.001; Figures 3C and 4A) and 1.5-fold greater in BA.1 BTIs compared with BA.1-infected unvaccinated individuals (p = 0.08; Figures 3D and 4B). While not significant for Delta-infected BTI, regardless of the infecting variant, BA.4 showed the biggest fold decrease of ADCC in both BTIs and unvaccinated but infected individuals (Figures 4A and 4B). For BA.1 BTIs, both BA.1 and BA.2 showed significantly higher responses compared with Beta, again suggesting significant antigenic differences between these variants in terms of ADCC, a pattern not noted for neutralization. Additionally, in contrast to neutralization, fold decreases of ADCC against VOCs relative to the infecting variant were similar in unvaccinated compared with vaccinated individuals (Figures 4C and 4D).

## Discussion

The ability of BA.4 to escape neutralization elicited by vaccination and previous infection is now well described.^6^^,^^31^^,^^32^^,^^33^^,^^43^^,^^44^^,^^45^^,^^46^^,^^47^^,^^48^^,^^49^ Here, we have extended these studies to define BA.4 resistance to neutralizing and FcγRIIIa signaling antibodies triggered by each of the four VOCs (D614G, Beta, Delta, and BA.1) that sequentially caused waves of infection in South Africa.^50^ Regardless of the infecting variant, we show that BA.4 shows particularly large reductions in neutralization for antibodies triggered by Delta and BA.1 compared with D614G and Beta. Secondly, we provide an assessment of the effect of BA.4 mutations on Fc effector function, which has been preserved against other VOCs.^16^^,^^51^ Using FcγRIIIa activation as a proxy for ADCC, we show that BA.4 shows greater ADCC escape than previous VOCs. As for neutralization, this loss is especially pronounced in Delta- and BA.1-infected individuals, including in BTIs. Our data extend previous studies to assess antibodies triggered by four VOCs and confirm that BA.4 shows reduced neutralization and ADCC regardless of the infecting variant.

Our data confirm our previous studies showing that VOCs trigger responses with different specificities.^18^^,^^35^ Here, the largest fold decreases for neutralization against BA.4 were seen for unvaccinated individuals previously infected with Delta and BA.1^44^. For Delta, this is in contrast to a previous report, where Delta-wave patient sera neutralized not only Delta but also the BA.4/5 and BA.2.12.1 variants, which, like Delta, contain substitutions at position L452.^46^ In our cohort, we noted that autologous titers to the Delta spike were higher than those in D614G and Beta, perhaps a consequence of the high viral loads that are associated with Delta infections.^52^ Of note, in BA.1 infections, we observed that neutralization of both BA.2 and BA.4 was reduced. However, BA.4 was more resistant than BA.2, despite the fact that these two sub-lineages of Omicron are genetically very similar.^30^ This suggests that BA.1-triggered antibodies may target epitopes including L452 and F486 that distinguish BA.2 and BA.4, which will form the basis of future mapping studies.

Although plasma shows substantial reduction in the ability to neutralize BA.4, we nonetheless observed relatively high titers in previously vaccinated individuals with BTIs. We and others have previously shown that BTIs result in significantly higher neutralization titers than in people who were not vaccinated prior to infection.^18^^,^^39^^,^^44^ High titers generally result in better neutralization of VOCs, which is the basis of ongoing booster regimens. However, we note that the fold loss in neutralization is higher in unvaccinated individuals compared with BTIs. This suggests that the preserved activity against VOCs such as BA.4 is not simply a consequence of higher starting titers but that the quality of nAbs resulting from BTIs is intrinsically better. This is consistent with ongoing affinity maturation or expansion of cross-reactive responses after second antigenic exposures.^53^ In South Africa, where >95% of people are now estimated to be seropositive, this scenario of hybrid immunity is likely very common.^38^^,^^54^ However, even in the context of these high titer responses, BA.4 shows reduced sensitivity to neutralization compared with other VOCs, perhaps accounting for ongoing community transmission.

This study provides an assessment of BA.4 mutations on Fc effector function. Here we show that BA.4 shows significant ADCC escape, which exceeds or equals that of previous VOCs. As for neutralization, this loss is especially pronounced in Delta- and BA.1-infected individuals, including in BTIs. This suggests that as for neutralization, the sequence of the infecting spike also affects the quality of antibodies mediating Fc effector function.^16^^,^^55^

ADCC and other Fc effector functions have proven to be remarkably resilient in the face of mutations in spike.^16^^,^^51^^,^^56^ However, our observation that BA.4 shows significantly reduced sensitivity to ADCC responses suggests limits to that tolerance and provides interesting insights into the targets of these antibodies. The observation that Beta-directed ADCC is most compromised following infection but not BTIs caused by Delta suggests differences in primary versus hybrid immunity, as well as in antibodies triggered by different VOCs. Furthermore, the reduced ADCC sensitivity of BA.4 suggests that regions mutated in this VOC may define key ADCC epitopes, which may or may not overlap with sites targeted in neutralization. Delineation of these sites will be key to defining the targets of antibodies mediating ADCC. However, ADCC and neutralization showed comparable cross-reactivity across the VOCs for each of the waves, with the Beta wave being the most cross-reactive, as we have previously shown.^16^^,^^36^ We further show compromised ADCC and neutralization of BA.1-infected plasma against D614G and Beta, as in our previous study.^18^ While patterns were similar across the waves, fold differences were dramatically reduced between variants for ADCC. Despite ADCC-mediating antibodies binding to more epitopes than those that neutralize, which are focused on several key sites such as the RBD, the antigenic landscapes of ADCC and neutralization are quite similar. These studies will be important in the assessment of Fc effector function against emerging VOCs and inform the development of universal vaccines for improved cross-reactivity against emerging VOCs.

Overall, these data extend previous studies to assess antibodies triggered by four VOCs and confirm that BA.4 escapes both neutralization and ADCC regardless of the infecting variant.^16^^,^^18^^,^^35^^,^^39^ The high level of resistance of BA.4, particularly to antibodies from BA.1 infections, provides an immunological mechanism for the rapid spread of BA.4 in South Africa immediately after a BA.1-dominated wave and provides insights into populational-level immunity gaps that may exist elsewhere. Furthermore, the reduced sensitivity of BA.4 to ADCC, unlike previous VOCs, provides useful insights for future mapping of the targets of antibodies mediating ADCC. Lastly, we note that although ADCC activity against BA.4 was reduced, residual activity may nonetheless contribute to the protection from severe disease. While T cells and other Fc effector functions not studied here almost certainly also contribute to this effect, the preserved ADCC against BA.4 is consistent with the observation of low levels of severe disease and hospitalization during this wave in South Africa.^57^

### Limitations of the study

We acknowledge that the numbers of individuals in several of these groups are small, and future studies should include additional donors. The gender ratio of cohorts varied by infecting variant, which may impact comparisons across cohorts. Similarly, time from infection differed between waves for both breakthrough and unvaccinated individuals as a result of difficulties in sampling. Samples that were obtained early post-infection may not have allowed for sufficient time to develop a BA.4 response, and future studies should focus on longitudinal samples. Additionally, not all samples were run across both ADCC and neutralization assays (as indicated in Table S1) as a result of sample availability. Furthermore, although we have extensive clinical follow up, we cannot rule out the possibility that convalescent donors experienced previous undocumented asymptomatic infection, which could alter the quality of humoral responses. For our studies of BTIs, these occurred after differing vaccine regimens, which may have affected humoral responses. We have not included measurements of T cell responses or Fc effector functions beyond ADCC to BA.4, including antibody-dependent cellular phagocytosis (ADCP) and antibody-dependent complement deposition (ADCD), which likely contribute to protection from severe disease. Lastly, viral sequences were available only for a sub-set of samples in each wave, though the samples were collected when each variant dominated infections during that particular wave.

## STAR★Methods

### Key resources table

**Table:** 

REAGENT or RESOURCE	SOURCE	IDENTIFIER
**Antibodies**		

CR3022	Genscript (https://www.genscript.com)	N/A
P2B-2F6	Dr Nicole Doria-Rose, VRC, USA	N/A
084-7D	This paper	N/A
AIRU946-E4	This paper	N/A
AIRU946-A6	This paper	N/A
anti-IgG APC (clone QA19A42)	Biolegend	Cat#366905; RRID:AB_2888847
Palivizumab	Medimmune	Synagis; RRID: AB_2459638

**Bacterial and virus strains**		

SARS-CoV-2 pseudoviruses for ancestral, Beta, Delta, Omicron BA.1, Omicron BA.2, Omicron BA.4	Wibmer et al.^1^; Richardson et al.,^16^ this paper	N/A

**Biological samples**		

Convalescent hospitalized blood samples	Groote Schuur Hospital	https://www.gsh.co.za
Convalescent hospitalized blood samples	Steve Biko Academic Hospital	https://www.sbah.org.za

**Chemicals, peptides, and recombinant proteins**		

SARS-CoV-2 original variant spike proteins	Original: Dr Jason McKellan	N/A

**Critical commercial assays**		

PEI-MAX 40,000	Polysciences	Cat # 24765-1
QUANTI-Luc luciferase	Invivogen	Cat# rep-qlc2
Luciferase	Promega	Cat# PRE263B-C

**Experimental models: Cell lines**		

Human Embryonic Kidney (HEK) 293F	Dr Nicole Doria-Rose, VRC, USA	N/A
HEK293T/ACE2.MF	Dr Michael Farzan, Scripps, USA	N/A
Jurkat-Lucia™ NFAT-CD16 cells	Invivogen	Cat # jktl-nfat-cd16
Human Embryonic Kidney (HEK) 293T cells	Dr George Shaw, UPenn,USA	N/A

**Recombinant DNA**		

Spike Hexapro plasmid	Original: Dr Jason McKellan Beta: Moyo-Gwete et al.^36^	N/A
SARS-CoV-2 ancestral variant spike (D614G) plasmid	Wibmer et al.^1^	N/A
Beta spike (L18F, D80A, D215G, K417N, E484K, N501Y, D614G, A701V, 242–244 del) plasmid	Wibmer et al.^1^	N/A
Delta spike (T19R, R158G L452R, T478K, D614G, P681R, D950N, 156–157 del) plasmid	Richardson et al.^16^	N/A
Omicron BA.1 plasmid (A67V, Δ69–70, T95I, G142D, Δ143-145, Δ211, L212I, 214EPE, G339D, S371L, S373P, S375F, K417N, N440K, G446S, S477N, T478K, E484A, Q493R, G496S, Q498R, N501Y, Y505H, T547K, D614G, H655Y, N679K, P681H, N764K, D796Y, N856K, Q954H, N969K, L981F)	Richardson et al.^16^	N/A
Omicron BA.2 plasmid (T19I, L24S, 25-27del, G142D, V213G, G339D, S371F, S373P, S375F, T376A, D405N, R408S,K417N, N440K, S477N, T478K, E484A, Q493R, Q498R, N501Y, Y505H, D614G, H655Y, N679K, P681H, N764K, D796Y, Q954H, N969K)	Richardson et al.^16^	N/A
Omicron BA.4 plasmid (T19I, L24S, Δ25-27, Δ69-70, G142D, V213G, G339D, S371F, S373P, S375F, T376A, D405N, R408S, K417N, N440K, L452R, S477N, T478K, E484A, F486V, Q498R, N501Y, Y505H, D614G, H655Y, N679K, P681H, N764K, D796Y, Q954H, N969K).	This paper	N/A
Firefly luciferase encoding lentivirus backbone plasmid	Dr Michael Farzan, Scripps	N/A

**Software and algorithms**		

FACSDiva 9	BD Biosciences	https://www.bdbiosciences.com
FlowJo 10	FlowJo, LLC	https://www.flowjo.com
Graphpad Prism 9	Graphpad	https://www.graphpad.com
Biorender	Biorender	https://www.biorender.com

### Resource availability

#### Lead contact

Further information and reasonable requests for resources and reagents should be directed to and will be fulfilled by the lead contact, Penny Moore (pennym@nicd.ac.za).

#### Materials availability

All unique/stable reagents generated in this study are available from the lead contact with a completed Materials Transfer Agreement.

### Experimental model and subject details

#### Human subjects

Plasma samples from the first SARS-CoV-2 wave (D614G-infected) were obtained from a previously described cohort across various sites in South Africa prior to September 2020.^1^ Second wave samples (Beta-infected) were obtained from a cohort of patients admitted to Groote Schuur Hospital, Cape Town in December 2020 - January 2021.^36^ Third wave samples (Delta-infected) were obtained from the Steve Biko Academic Hospital, Tshwane from patients admitted in July 2021.^35^ Samples infected in the fourth COVID-19 wave of infection in South Africa were collected from participants enrolled to the Pretoria COVID-19 study cohort. Participants were admitted to Tshwane District Hospital (Pretoria, South Africa) between 25 November 2021- 20 December 2021 (Table S1). In all waves, samples were collected when more than 90% of SARS-CoV-2 cases in South Africa were caused by the respective variants. Sequence confirmation was only available for a subset of samples but all the samples that were sequenced corresponded to the appropriate variant for that wave. All samples were from HIV-negative individuals who were above 18 years of age and provided consent. Ethical clearance was obtained for each cohort from Human Research Ethics Committees from the University of Pretoria (247/2020) and University of Cape Town (R021/2020). All participants had PCR-confirmed SARS-CoV-2 infection before blood collection Written informed consent was obtained from all participants. BTI participants were recruited from HCWs at the NICD, Steve Biko Academic Hospital (Tshwane, South Africa) and Groote Schuur Hospital (Cape Town, South Africa). Lack of prior infection in these individuals was confirmed by Nucleocapsid ELISA.

#### Cell lines

Human embryo kidney HEK293T cells were cultured at 37°C, 5% CO_2_, in DMEM containing 10% heat-inactivated fetal bovine serum (Gibco BRL Life Technologies) and supplemented with 50 μg/mL gentamicin (Sigma). Cells were disrupted at confluence with 0.25% trypsin in 1 mM EDTA (Sigma) every 48–72 h. HEK293T/ACE2.MF cells were maintained in the same way as HEK293T cells but were supplemented with 3 μg/mL puromycin for selection of stably transduced cells. HEK293F suspension cells were cultured in 293 Freestyle media (Gibco BRL Life Technologies) and cultured in a shaking incubator at 37°C, 5% CO_2_, 70% humidity at 125rpm and maintained between 0.2 and 0.5 million cells/mL. Jurkat-Lucia NFAT-CD16 cells were maintained in IMDM media with 10% heat-inactivated fetal bovine serum (Gibco, Gaithersburg, MD), 1% Penicillin Streptomycin (Gibco, Gaithersburg, MD) and 10 μg/mL of Blasticidin and 100 μg/mL of Zeocin was added to the growth medium every other passage. Cells were cultured at 37°C, 5% CO_2_ in RPMI containing 10% heat-inactivated fetal bovine serum (Gibco, Gaithersburg, MD) with 1% Penicillin Streptomycin (Gibco, Gaithersburg, MD) and 2-mercaptoethanol to a final concentration of 0.05 mM and not allowed to exceed 4 × 10^5^ cells/mL to prevent differentiation.

### Method details

#### Spike plasmid and lentiviral pseudovirus production

The SARS-CoV-2 ancestral strain spike, cloned into pCDNA3.1 was mutated using the QuikChange Lightning Site-Directed Mutagenesis kit (Agilent Technologies) and NEBuilder HiFi DNA Assembly Master Mix (NEB) to include D614G (original) or lineage defining mutations for Beta (L18F, D80A, D215G, 242-244del, K417N, E484K, N501Y, D614G and A701V), Delta (T19R, 156-157del, R158G, L452R, T478K, D614G, P681R and D950N), Omicron BA.1 (A67V, Δ69–70, T95I, G142D, Δ143-145, Δ211, L212I, 214EPE, G339D, S371L, S373P, S375F, K417N, N440K, G446S, S477N, T478K, E484A, Q493R, G496S, Q498R, N501Y, Y505H, T547K, D614G, H655Y, N679K, P681H, N764K, D796Y, N856K, Q954H, N969K, L981F), Omicron BA.2 (T19I, L24S, 25-27del, G142D, V213G, G339D, S371F, S373P, S375F, T376A, D405N, R408S,K417N,N440K, S477N, T478K, E484A, Q493R, Q498R, N501Y, Y505H, D614G, H655Y, N679K, P681H, N764K, D796Y, Q954H, N969K) or Omicron BA.4 (T19I, L24S, Δ25-27, Δ69-70, G142D, V213G, G339D, S371F, S373P, S375F, T376A, D405N, R408S, K417N, N440K, L452R, S477N, T478K, E484A, F486V, Q498R, N501Y, Y505H, D614G, H655Y, N679K, P681H, N764K, D796Y, Q954H, N969K).

Pseudotyped lentiviruses were prepared by co-transfecting HEK293T cell line with the SARS-CoV-2 ancestral variant spike (D614G), Beta, Delta, C.1.2, Omicron BA.1 or Omicron BA.2 spike plasmids in conjunction with a firefly luciferase encoding lentivirus backbone (HIV-1 pNL4.luc) plasmid. Culture supernatants were clarified of cells by a 0.45-μM filter and stored at −70°C.

#### SARS-CoV-2 antigens

For serology, SARS-CoV-2 spike variant proteins with S2P mutations were expressed in Human Embryonic Kidney (HEK) 293F suspension cells by transfections. After incubating for six days at 37°C, 70% humidity and 10% CO2, proteins were first purified using a nickel resin followed by size-exclusion chromatography. Relevant fractions were collected and frozen at −80°C until use.

#### SARS-CoV-2 spike enzyme-linked immunosorbent assay (ELISA)

Two μg/mL of spike protein were used to coat 96-well, high-binding plates and incubated overnight at 4°C. The plates were incubated in a blocking buffer consisting of 5% skimmed milk powder, 0.05% Tween 20, 1x PBS. Plasma samples were diluted to 1:100 starting dilution in a blocking buffer and added to the plates. IgG secondary antibody was diluted to 1:3000 in blocking buffer and added to the plates followed by TMB substrate (Thermofisher Scientific). Upon stopping the reaction with 1 M H2SO4, absorbance was measured at a 450nm wavelength. In all instances, mAbs CR3022 and AIRU946-A6 were used as positive controls and Palivizumab was used as a negative control.

#### Pseudovirus neutralization assay

For the neutralization assay, plasma samples were heat-inactivated and clarified by centrifugation. Heat-inactivated plasma samples were incubated with the SARS-CoV-2 pseudotyped virus for 1 h at 37°C, 5% CO_2_. Subsequently, 1 × 10^4^ HEK293T cells engineered to over-express ACE-2 (293T/ACE2.MF)(kindly provided by M. Farzan (Scripps Research)) were added and incubated at 37°C, 5% CO_2_ for 72 h upon which the luminescence of the luciferase gene was measured. Titers were calculated as the reciprocal plasma dilution (ID_50_) causing 50% reduction of relative light units. CB6 and CA1 were used as positive controls for D614G, Beta and Delta. 084-7D, a mAb targeting K417N was used as a positive control for Omicron BA.1 and Beta. Samples were run in duplicate and repeated a minimum of two times.

#### FcγRIIIa (CD16) signaling assay

The ability of plasma antibodies to cross-link and signal through FcγRIIIa (CD16) and spike expressing cells was measured as a proxy for ADCC. For spike assays, HEK293T cells were transiently transfected with 5μg of native SARS-CoV-2 spike plasmids using PEI-MAX 40,000 (Polysciences) and incubated for 2 days at 37°C. AIRU946-A6, which binds to different soluble spike variants comparably as determined by ELISA (Figure S2A), was used to confirm similar amounts of spike expression on the surface of the cells across variants (Figures S2B and S2C) through the detection by anti-IgG PE staining measured by flow cytometry. Palivizumab against all variants, untransfected cells and transfected cells not incubated with mAb were used as negative controls (Figures S2C and S2D). Subsequently, 1 × 10^5^ spike transfected cells per well were incubated with heat inactivated plasma (1:100 final dilution) or monoclonal antibodies (final concentration of 100 μg/mL) in RPMI 1640 media supplemented with 10% FBS 1% Pen/Strep (Gibco, Gaithersburg, MD) for 1 h at 37°C. Jurkat-Lucia NFAT-CD16 cells (Invivogen) (2 × 10^5^ cells/well and 1 × 10^5^ cells/well for spike and other protein respectively) were added and incubated for 24 h at 37°C, 5% CO_2_. Twenty μL of supernatant was then transferred to a white 96-well plate with 50 μL of reconstituted QUANTI-Luc secreted luciferase and read immediately on a Victor 3 luminometer with 1s integration time. Normalised relative light units (RLU) of a no antibody control was subtracted as background. Palivizumab was used as a negative control, while CR3022 was used as a positive control, and P2B-2F6 to differentiate the Beta from the D614G variant. 084-7D was used as a positive control for Omicron BA.1 and Beta. AIRU946-E4 and AIRU946-A6 were used as additional positive controls showing similar activity across variants. A positive threshold was set using 10 SARS-CoV-2 negative plasma samples from prior to the pandemic (Figure S3). To induce the transgene 1x cell stimulation cocktail (Thermofisher Scientific, Oslo, Norway) and 2 μg/mL ionomycin in R10 was added as a positive control to confirm sufficient expression of the Fc receptor. RLUs for spikes were normalised to each other and between runs using AIRU946-A6. All samples were run head to head in the same experiment as were all variants tested. Samples were run in duplicate and repeated a minimum of two times.

### Quantification and statistical analysis

Analyses were performed in Prism (v9; GraphPad Software Inc, San Diego, CA, USA). Non-parametric tests were used for all comparisons. The Friedman test with Dunns correction for multiple comparisons was used for matched comparisons across variants. All correlations reported are non-parametric Spearman’s correlations. *p* values less than 0.05 were considered to be statistically significant.

## Data Availability

• All data reported in this paper will be shared by the lead contact upon request. • This paper does not report original code. • Any additional information required to reanalyze the data reported in this paper is available from the lead contact upon request.

## Supplemental information

Document S1. Figures S1–S3 and Table S1

Document S2. Article plus supplemental information
