# Supplementary material for: Antibody-dependent cellular cytotoxicity against SARS-CoV-2 Omicron sub-lineages is reduced in convalescent sera regardless of infecting variant
Source: Cell Rep Med. 2022 Dec 22;4(1):100910. doi: 10.1016/j.xcrm.2022.100910 (PMC9771750; doi:10.1016/j.xcrm.2022.100910)
Supplement: Document S1. Figures S1–S3 and Table S1 [file mmc1.pdf]

Cell Reports Medicine, Volume *4*

Supplemental information

Antibody-dependent cellular cytotoxicity against SARS-CoV-2 Omicron sub-lineages is reduced in convalescent sera regardless of infecting variant

Simone I. Richardson, Prudence Kgagudi, Nelia P. Manamela, Haajira Kaldine, Elizabeth M. Venter, Thanusha Pillay, Bronwen E. Lambson, Mieke A. van der Mescht, Tandile Hermanus, Sashkia R. Balla, Zelda de Beer, Talita R. de Villiers, Annie Bodenstein, Gretha van den Berg, Marizane du Pisanie, Wendy A. Burgers, Ntobeko A.B. Ntusi, Fareed Abdullah, Veronica Ueckermann, Theresa M. Rossouw, Michael T. Boswell, and Penny L. Moore

Table S1: Demographic and clinical information related to STAR Methods

Patient ID	Infecting variant by date or confirmed by sequence	Age range	Gender	Positive PCR test	Days sampled post infection	Vaccination status	WHO severity scale	Tested in ADCC	Tested in Binding	Tested in neutralization
COV004	D614G	60-64	Male	2020/05/23	1	Unvaccinated	4	Y	Y	Y
COV006	D614G	25-29	Male	2020/05/25	3	Unvaccinated	3	Y	Y	Y
COV020	D614G	50-54	Male	2020/06/09	3	Unvaccinated	6	Y	Y	Y
COV021	D614G	45-50	Female	2020/06/11	1	Unvaccinated	4	Y	Y	Y
COV024	D614G	65-69	Male	2020/06/12	1	Unvaccinated	4	Y	Y	Y
COV025	D614G	45-49	Female	2020/06/24	9	Unvaccinated	4	Y	Y	Y
COV036	D614G	40-44	Male	2020/06/27	2	Unvaccinated	8	Y	Y	Y
COV042	D614G	35-39	Male	2020/07/18	4	Unvaccinated	4	Y	Y	Y
COV043	D614G	50-54	Male	2020/07/19	6	Unvaccinated	5	Y	Y	Y
CoV044	D614G	45-49	Male	2020/08/07	3	Unvaccinated	5	Y	Y	Y
34593520	D614G	Unknown	Unknown	Unknown	Unknown	Unvaccinated	2	N	N	Y
34578424	D614G	Unknown	Unknown	Unknown	Unknown	Unvaccinated	2	N	N	Y
34587804	D614G	Unknown	Unknown	Unknown	Unknown	Unvaccinated	2	N	N	Y
SO3357/20	D614G	Unknown	Unknown	Unknown	Unknown	Unvaccinated	2	N	N	Y
SO4681/20	D614G	Unknown	Unknown	Unknown	Unknown	Unvaccinated	2	N	N	Y
34647641	D614G	Unknown	Unknown	Unknown	Unknown	Unvaccinated	2	N	N	Y
SA-01-0018	Beta	60-65	Male	2020/12/07	29	Unvaccinated	4	Y	Y	Y
SA-01-0025	Beta	56-59	Female	2020/12/22	14	Unvaccinated	4	Y	N	Y
SA-01-0040	Beta	75-79	Male	2021/01/04	3	Unvaccinated	4	N	N	Y
SA-01-0042	Beta	40-44	Female	2020/12/09	29	Unvaccinated	4	Y	Y	Y
SA-01-0075	Beta	75-79	Male	2021/01/03	10	Unvaccinated	4	Y	Y	Y
SA-01-0081	Beta	65-69	Female	2020/12/30	14	Unvaccinated	4	Y	Y	Y
SA-01-0032	Beta	65-69	Female	2020/12/23	14	Unvaccinated	4	Y	Y	Y
SA-01-0038	Beta	50-54	Male	2020/12/25	13	Unvaccinated	4	Y	Y	Y
SA-01-0052	Beta	65-69	Female	2021/01/03	5	Unvaccinated	4	Y	Y	Y
SA-01-0068	Beta	35-39	Male	2020/12/23	20	Unvaccinated	4	N	N	Y
CoV113_V1	Delta	65-69	Female	2021/07/09	11	Unvaccinated	3	Y	N	Y
CoV114_V1	Delta	65-69	Male	2021/07/12	3	Unvaccinated	3	Y	Y	Y
CoV116_V1	Delta	65-69	Female	2021/07/17	5	Unvaccinated	3	Y	Y	Y
CoV125_V1	Delta	85-89	Male	2021/07/15	1	Unvaccinated	3	Y	Y	Y
COV109 V1	Delta	30-34	Female	2021/07/13	1	Unvaccinated	3	N	Y	Y
COV111 V1	Delta	50-54	Female	2021/07/08	6	Unvaccinated	3	N	Y	Y
COV118 V1	Delta	45-49	Female	2021/07/19	1	Unvaccinated	3	N	Y	Y
SA-0-0157	Delta	60-64	Male	2021/07/13	1	Unvaccinated	1	Y	Y	N
SA-01-0140	Delta	40-44	Female	2021/07/07	1	Unvaccinated	2	Y	Y	N
SA-01-0154	Delta	30-34	Female	2021/07/05	9	Unvaccinated	2	Y	Y	N
SA-01-0155	Delta	75-79	Female	2021/07/08	6	Unvaccinated	2	Y	Y	N
SA-01-0159	Delta	35-39	Male	2021/07/09	6	Unvaccinated	2	Y	Y	N
SA-01-0162	Delta	40-44	Female	2021/07/09	6	Unvaccinated	2	Y	Y	N
CoVBT001	Delta	25-29	Female	2021/06/15	30	One dose Ad26.CoV2.S	2	N	Y	Y
CoVBT002	Delta	25-29	Female	2021/06/21	30	One dose Ad26.CoV2.S	1	N	Y	Y
CoVBT003	Delta	35-39	Female	2021/06/19	30	One dose Ad26.CoV2.S	2	N	Y	Y
CoVBT004	Delta	40-44	Female	2021/06/25	30	One dose Ad26.CoV2.S	2	N	Y	Y
CoVBT005	Delta	50-54	Female	2021/07/06	30	One dose Ad26.CoV2.S	2	N	Y	Y
CoVBT006	Delta	25-29	Female	2021/06/02	30	One dose Ad26.CoV2.S	2	N	Y	Y
CoVBT007	Delta	25-29	Female	2021/07/21	30	One dose Ad26.CoV2.S	1	N	Y	Y
CoVBT008	Delta	35-39	Female	2021/07/16	30	One dose Ad26.CoV2.S	2	N	Y	Y
CoVBT009	Delta	40-44	Female	2021/07/07	30	One dose Ad26.CoV2.S	2	N	Y	Y
CoVBT010	Delta	25-29	Female	2021/07/16	30	One dose Ad26.CoV2.S	2	N	Y	Y
CoVBT011	Delta	40-44	Male	2021/07/01	30	One dose Ad26.CoV2.S	1	N	Y	Y
CoVFU082	Delta	50-54	Female	2021/06/28	30	One dose Ad26.CoV2.S	2	Y	Y	Y
CoVFU086	Delta	60-64	Female	2021/06/24	30	One dose Ad26.CoV2.S	2	Y	Y	Y
CoVFU088	Delta	40-44	Female	2021/06/28	30	One dose Ad26.CoV2.S	2	Y	Y	Y
CoVFU094	Delta	40-44	Female	2021/07/09	30	One dose Ad26.CoV2.S	2	Y	Y	Y
CoVFU041	Delta	60-64	Female	2021/07/16	30	One dose Ad26.CoV2.S	1	Y	Y	Y
CoVBT946	Delta	30-34	Female	2021/08/05	30	One dose Ad26.CoV2.S	2	Y	Y	Y
COV132	Omicron BA.1	36-39	Male	2021/11/28	9	Unvaccinated	6	Y	Y	Y
COV140	Omicron BA.1	25-29	Female	2021/12/09	1	Unvaccinated	2	Y	Y	Y
COV145	Omicron BA.1	15-19	Female	2021/12/03	3	Unvaccinated	2	Y	Y	Y
COV149	Omicron BA.1	60-64	Female	2021/12/05	3	Unvaccinated	2	Y	Y	Y
COV157	Omicron BA.1	65-69	Male	2021/12/04	4	Unvaccinated	5	Y	Y	Y
COV158	Omicron BA.1	65-69	Female	2021/12/14	6	Unvaccinated	5	Y	Y	Y
COV159	Omicron BA.1	20-24	Male	2021/11/25	6	Unvaccinated	2	Y	Y	Y
COV162	Omicron BA.1	55-59	Male	2021/12/09	5	Unvaccinated	5	Y	Y	Y
COV165	Omicron BA.1	60-64	Female	2021/12/10	4	Unvaccinated	5	Y	Y	Y
COV166	Omicron BA.1	55-59	Male	2021/12/10	4	Unvaccinated	6	Y	Y	Y
COV167	Omicron BA.1	60-64	Female	2021/11/29	8	Unvaccinated	5	Y	Y	Y
COV176	Omicron BA.1	75-79	Male	2021/12/20	2	Unvaccinated	6	Y	Y	Y
COV178	Omicron BA.1	60-64	Female	2021/12/19	2	Unvaccinated	5	Y	Y	Y
COV183	Omicron BA.1	25-29	Male	2021/12/12	2	Unvaccinated	2	Y	Y	Y
COV184	Omicron BA.1	55-59	Male	2021/12/14	1	Unvaccinated	5	Y	Y	Y
COV185	Omicron BA.1	45-49	Female	2021/12/13	1	Unvaccinated	5	Y	Y	Y
COV186	Omicron BA.1	55-59	Male	2021/12/03	10	Unvaccinated	5	Y	Y	Y
COV187	Omicron BA.1	35-39	Female	2021/12/11	3	Unvaccinated	2	Y	Y	Y
COV189	Omicron BA.1	45-49	Male	2021/12/09	5	Unvaccinated	5	Y	Y	Y
COV190	Omicron BA.1	30-34	Female	2021/12/10	4	Unvaccinated	2	Y	Y	Y
COV182	Omicron BA.1	35-39	Male	2021/12/10	3	One dose Ad26.CoV2.S	2	Y	Y	Y
COV131	Omicron BA.1	55-59	Female	2021/12/10	4	Two doses BNT162b2	6	Y	Y	Y
COV135	Omicron BA.1	85-89	Male	2021/11/26	5	Two doses BNT162b2	2	Y	Y	Y
COV138	Omicron BA.1	65-69	Male	2021/11/25	5	Two doses BNT162b2	3	Y	Y	Y
COV161	Omicron BA.1	60-64	Female	2021/12/05	5	Two doses BNT162b2	2	Y	Y	Y
COV181	Omicron BA.1	70-74	Male	2021/12/16	4	Two doses BNT162b2	6	Y	Y	Y

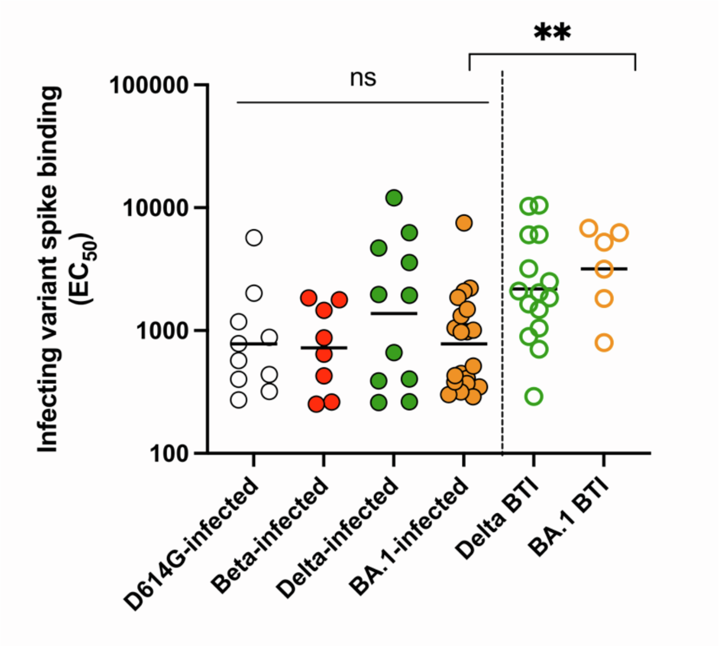

Figure S1: Binding responses against the infected variant spike across different infection waves and previously vaccinated samples, related to Figure 1 and 2
Levels of binding antibodies (represented as EC_{50}) against respective infecting spikes as measured by ELISA are shown. Significance across infection waves was calculated using a Kruskal-Wallis test while comparisons between BA.1 and Delta unvaccinated and previously vaccinated but breakthrough infected (BTI) comparisons were done using the Mann-Whitney t test where **$p<0.001$. Lines represent the geometric mean of the groups as determined by two independent experiments while the dotted line separates unvaccinated from previously vaccinated samples.

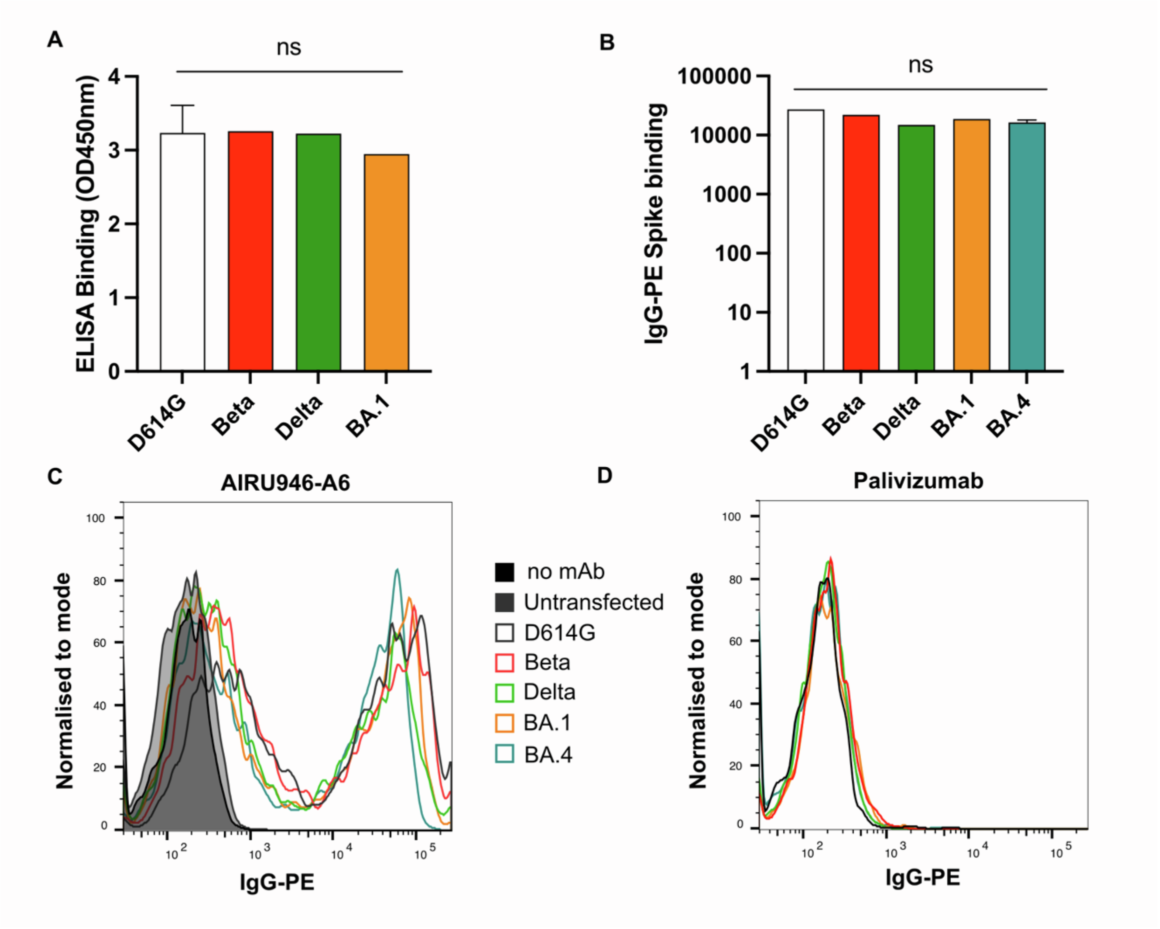

Figure S2: Spike expression levels of different SARS CoV-2 variants on HEK293T cells, related to Figure 3 and 4.

MAb AIRU946-A6 was tested for binding to (A) soluble spike proteins by ELISA and (B) spike transfected HEK293T cells of D614G, Beta, Delta and BA.1 by flow cytometry represented as the proportion of IgG positive cells multiplied by the geometric mean intensity of that population. Significance was calculated using an ANOVA and bars represent the mean of two independent experiments, with error bars representing the standard deviation. Flow cytometry histograms of (C) positive control AIRU946-A6 and (D) negative control Palivizumab binding to untransfected (grey) or variant spike transfected cells as detected by anti-IgG-PE.

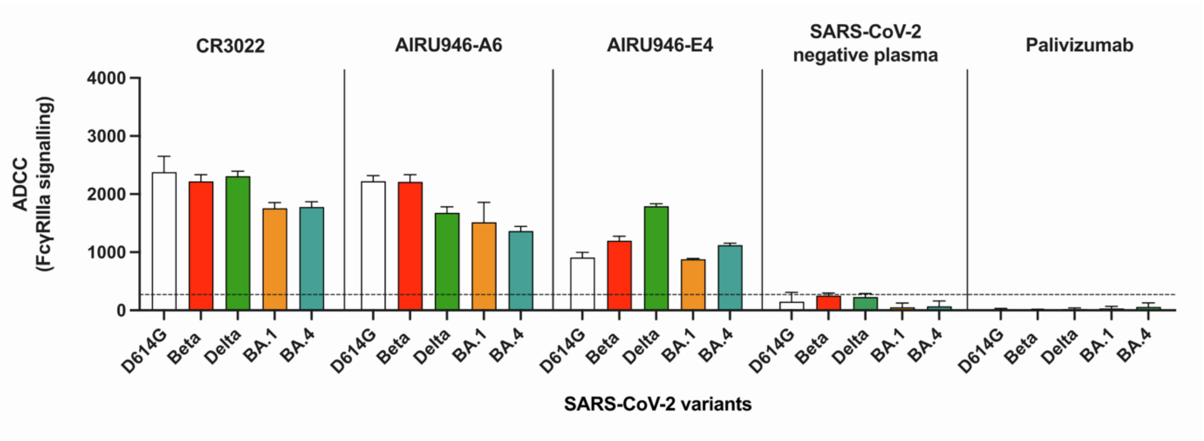

Figure S3: FcγRIIIa signalling assay controls and positivity threshold, related to Figure 3 and 4.
The ability of mAbs and pre-pandemic plasma to perform FcγRIIIa signalling as a proxy for ADCC against D614G (white), Beta (red), Delta (green), BA.1(orange) and BA.4 (blue) spike transfected cells is shown. mAbs CR3022, AIRU946-A6, and AIRU946-E4 were positive controls with Palivizumab and 10 SARS-CoV-2 negative (pre-pandemic) plasma samples used to set a threshold of positivity as indicated by the dotted line. These data represent two independent experiments with bars representative of the mean and error bars of the standard deviation.
